# Airway epithelial Paraoxonase-2 in obese asthma

**DOI:** 10.1371/journal.pone.0261504

**Published:** 2022-03-14

**Authors:** Daniel Efrain Winnica, Anthony Monzon, Shuyu Ye, Eszter K. Vladar, Maxwell Saal, Riley Cooney, Cuining Liu, Sunita Sharma, Fernando Holguin

**Affiliations:** Department of Medicine, Division of Pulmonary Science and Critical Care, School of Medicine, University of Colorado, Aurora, Colorado, United States of America; B. S. Abdur Rahman Crescent Institute of Science and Technology, INDIA

## Abstract

**Background:**

Obesity in asthmatics has been associated with higher airway oxidative stress in which dysfunctional mitochondria are a potential contributing source of excess free radicals. Paraoxonase 2 (PON2) plays an important role in reducing mitochondrial-derived oxidative stress and could, therefore, have therapeutic potential in these patients.

**Objectives:**

We used primary human bronchial epithelial cells (HBECs) from asthmatics and healthy controls to evaluate: a) protein levels of Paraoxonase 2 and b) to test the potential protective effect of quercetin supplementation in cells under oxidative stress conditions.

**Results:**

Compared to lean controls, obese asthmatics had significantly lower PON2 airway epithelial levels (respectively, 1.08 vs. 0.47 relative units normalized by GAPDH) (p-value < 0.006). Treating HBECs in vitro for 24 hrs. with 25μM quercetin significantly increased PON2 protein levels: 15.5 treated cells vs. 9.8 untreated cells (relative units normalized by GAPDH) (p value = 0.004). Notably, compared to untreated cells, quercetin supplementation reduces mitochondrial superoxide and hydrogen peroxide production on HBECs cells exposed to different oxidative stress triggers such as 1–2 Naphthoquinone (1–2 NQ) and hydrogen peroxide, suggesting that PON2 might play a protective role ameliorating oxidative injury on human airway epithelium.

**Conclusion:**

Compared to lean controls, obese asthmatics have significantly reduced PON2 levels in airway epithelial cells. Treatment with quercetin in vitro increased PON2 protein levels and prevented oxidative stress from different types of stimuli. Hence, quercetin supplementation may be a potential therapeutic strategy to prevent obesity-mediated airway oxidative stress in obese asthmatics.

## Introduction

In patients with asthma, obesity and weight gain have been associated with increased asthma severity, which is a significant public health problem, given that nearly 40% of asthmatics in the United States are obese [1]. In obese asthmatics, mitochondrial dysfunction has been shown to contribute to airway oxidative stress, which could explain why inhaled corticosteroid lack efficacy in this subgroup of patients [2–6]. Mitochondrial structural and functional abnormalities have been shown to occur in the airway epithelium of asthmatics and to correlate with disease severity [7, 8]; these changes have also been induced in experimental animal models of asthma [9, 10]. Mitochondrial-derived oxidative reactive oxygen species (mtROS) are generated during the transportation of electrons in the tricarboxylic acid cycle. Under normal conditions, small amounts of reactive oxygen species (ROS) that leak out from the mitochondrial electron transport chain (ETC) can be generally handled by cellular defense mechanisms [11–14]. However, under pathologic conditions, such as asthma and obesity, antioxidant defense mechanisms may not be sufficient to prevent increased mtROS from injuring the cell and further worsening mitochondrial dysfunction.

The intracellular protein PON2 is confined to the mitochondrial linked to Complex III; it has been reported that the absence of or decreased levels of it led to mitochondrial malfunctioning [15]. The primary localization of PON2 in the mitochondria could support a role for this enzyme in protecting cells from mtROS derived oxidative damage. Given that PON2 reduces oxidative stress-mediated toxicity, it is possible that increasing its levels in the airway epithelial cells could provide therapeutic benefits to subjects with obesity and asthma [16–21].

Quercetin (3, 3’, 4’, 5, 7-pentahydroxyflavanone), is a naturally occurring flavonoid polyphenol, which is present in several plants and fruits; it can regulate mitochondrial redox status, potentially preventing or delaying disease progression, and improving mitochondrial function in both *in vitro* and *in vivo* experiments [22]. These effects could be partially explained by quercetin’s capacity to upregulate expression *in vitro* and increase levels of PON2 in different cell types [23, 24]. The therapeutic potential of quercetin has been highlighted in a recent study showing that it fully restores corticosteroid sensitivity in peripheral blood mononuclear cells (PBMCs) from Chronic Obstructive Pulmonary Disease (COPD) patients and in human monocytes (U937) exposed to cigarette smoke extract [25, 26]. We used Human Bronchial Epithelial BEAS-2B cells and primary Human Bronchial Epithelial Cells (HBECs) from asthmatics and healthy controls to test our hypotheses that increasing PON2 expression protects the airway epithelial cells from increased oxidative stress.

## Material and methods

### Materials

Bronchial Epithelial Cell Basal Medium was from Clonetics cat #CC-3171, fetal bovine serum (FBS), and Hank’s balanced salt solution (HBSS) were from Invitrogen (Carlsbad, CA). The anti-PON2 antibody was obtained from Abcam (Cambridge, MA, USA), ab96553, rabbit polyclonal, 1/1000 final dilution. Dimethylsulfoxide (DMSO) and 1,2-naphthoquinone (1,2 NQ) were from Sigma-Aldrich (St Louis, MO), hydrogen peroxide (H_2_O_2_) was from ACROS Organic, goat anti-GAPDH antibody was from Novus Biological, NB300-320, polyclonal, final concentration 0.05ug/mL, and 2-(3,4-dihydroxy phenyl)-3,5,7-trihydroxy-4H-1-benzopyran-4-one (Quercetin; >95%), was from Sigma-Aldrich (St. Louis, MO); 2,3-Dimethoxy-1,4-naphthoquinone (DMNQ), was from AdipoGen, Life Sciences. MitoSOX^™^ Red Mitochondrial Superoxide Indicator (M 36008), was from Invitrogen, Amplex^™^ Red Hydrogen Peroxide/Peroxidase Assay Kit (A22188), was from TermoFisher Scientific and AllPrep DNA/RNA/miRNA Universal Kit (cat. no. 80224) was from QIAGEN.

### Human subjects

The study population for the HBECs studies was derived from healthy volunteers and from asthmatics undergoing a baseline bronchoscopy approved by University of Pittsburgh,”Electrophilic Fatty Acid Derivatives in Asthma” (EFADA), study (IRB#: RO11010186) and approved by Colorado Multiple Institutional Review Board, University of Colorado, “Obesity, Metabolic Dysregulation and the Airway Epithelium in Asthmatics” study (COMIRB#16–2522). Participants signed a written consent form and agreed to undergo bronchoscopy using an IRB approved conscious sedation protocol. All asthmatics had physician-diagnosed asthma, mild to moderate disease, and had either a significant (> 12%) bronchodilator response or a ≥ 20% drop from baseline in the forced exhalation volume in the first second (FEV1) after a methacholine challenge.

### Mitochondrial reactive oxygen species production

We measured mitochondrial superoxide production using Mitosox, as previously described [27]. Mitosox Red reagent is a fluorogenic dye specifically targeted to mitochondria in live cells, it is quickly oxidized by superoxide but not by reactive nitrogen species (RNS) nor other reactive oxygen species (ROS). Briefly, cells were incubated with Mitosox reagent, 5 μM for ten minutes and the fluorescent intensity of the oxidized product (510ex/580em nm) was measured and normalized to cell number.

### Hydrogen peroxide production

To detect hydrogen peroxide (H_2_O_2_), we used 10-acetyl-3,7-dihydroxyphenoxazine (Amplex^®^ Red reagent) in combination with horseradish peroxidase (HRP). The Amplex^®^ Red reagent reacts with H_2_O_2_ in a 1:1 stoichiometry to produce the red-fluorescent oxidation product, resorufin, which has excitation maxima of 530 nm and emission maxima of 590 nm. Briefly, culture medium from the upper chamber was removed on day twenty-one and washed twice with 200μl PBS, and then 100μl of Phosphate Buffer Saline (PBS) was added following 3h incubation at 37°C H_2_O_2_ was then measured in the upper chamber supernatants using the Amplex Red reagent. The fluorescent signal was read at 530nm excitation, 590nm emissions, using the Infinite 200 PRO (Tecan, Männedorf, Switzerland).

### Cell culture

Human airway epithelial cells samples were obtained during bronchoscopies using the Severe Asthma Research Protocol (SARP), as previously described [28]. Briefly, participants with asthma and healthy controls underwent bronchoscopy using an IRB approved conscious sedation protocol after signing informed consent. A total of 5 cytology brushings were obtained from third or fourth airway generation branches from different subsegments of the right lung. For primary human bronchial epithelial cell (HBEC) culture, epithelial cells were placed directly into 10 mL of ice-cold PBS, centrifuged, washed, and resuspended in 1 mL of serum-free, hormonally supplemented bronchial epithelial growth medium (Clonetics, San Diego, Calif) containing 50 μg/mL gentamicin and 50 μg/mL amphotericin. A total of 4 × 10^5^ cells were seeded into 60-mm tissue-culture dishes coated with rat-tail type I collagen (BD Discovery Labware, Bedford, Mass). Cells were cultured at 37°C in a 5% CO2 environment. When the epithelial cells reached 70% to 80% confluence, they were dissociated with trypsin-EDTA and passed onto collagen-coated polyester transwell inserts of 12 mm in diameter (pore size, 0.4 μm) at 4 × 10^4^ cells/cm^2^. After a week of being in the immersed culture, epithelial cells reached 100% confluence and were shifted to an air-liquid interface (ALI) condition by removing all apical medium. Cell media was changed every other day. Cells were maintained for 21 days to differentiate into ciliated, mucus-producing cells [29]. Fresh medium was provided every 48 hours and 24 hours before harvest. Cells were then treated with quercetin 25 μM and 50μM.

Human bronchial epithelial cells (BEAS2B) were purchased from the American Type Culture Collection (ATCC) cultured in serum-free, hormonally supplemented bronchial epithelial growth medium (Clonetics; San Diego, CA, USA) containing 50 μg/mL gentamicin and 50 μg/mL amphotericin, and maintained at 37°C in a humidified atmosphere with 5% CO2/95% air.

### PON2 immunolabeling in primary human bronchial epithelial cells (HBECs)

Fully differentiated HBEC cultures were fixed in -20°C methanol for 10 min, blocked in 10% normal horse serum and 0.1% Triton X-100 in PBS and incubated with primary antibodies (anti-PON2 ab96553, and anti-COX IV ab62164) overnight at 4°C, then with Alexa dye conjugated secondary antibodies (Thermo Fisher) for 30 min at room temperature. Samples were mounted in Mowiol mounting medium containing 2% N-propyl gallate (Sigma) and imaged on a Zeiss LSM 900 confocal microscope (Zeiss).

### RNA sequencing

Total RNA from human airway epithelial cells were purified (AllPrep DNA/RNA/miRNA Universal Kit; QIAGEN, Hilden, Germany). Total RNA obtained were submitted to the Genomics Shared Resource at the School of Medicine, University of Colorado-Anschutz for library preparation and sequencing (Tecan Universal Plus mRNASEQ kit, Redwood City, CA, USA; Illumina NovaSeq6000, San Diego, CA, USA). Illumina adaptor sequence and low quality sequencing bases were removed from RNA-sequencing reads using cutadapt [30]. The resulting reads were aligned to the human genome (GRCh38.p13, GENCODE [31] release 37) using STAR [32], allowing us to quantify participants’ expression levels of genes, including PON2. We used DESeq2 [33] to normalize counts (VST-normalization; accounts for factors like differing total RNA library size between samples) counts and test for differential expression (Wald test). After normalization, the number of reads aligned to the PON2 gene are proportional to the number of transcripts of the gene detected in the sample.

### Transfection of PON2 small interfering RNA (siRNA)

To suppress PON2 expression, Human bronchial epithelial cells (BEAS2B) were transfected with 50 nM Silencer Select^™^ siRNA (s10834) (Invitrogen, Carlsbad, CA, USA) using Lipofectamine 2000 kit from Invitrogen according to the manufacturer’s recommended protocol. One day before transfection, BEAS2B cells were seeded at a density of 5.0× 10^5^ cells on 30 mm plates in growth medium without antibiotics. At 30–50% confluence, cells were transfected with PON2 siRNA or scrambled siRNA. The medium was changed after 6 h. The cells were incubated for 24–48 h at 37°C in a humidified incubator with 5% CO2.

### Cytotoxicity

It was performed using the Pierce LDH Cytotoxicity assay kit according to the manufacturer. Briefly, the assay was made by transferring cell culture media from treated and untreated cells into a microplate and adding the kit reagents. After incubation at room temperature for 30 minutes, reactions were stopped, and LDH activity was determined by spectrophotometric absorbance at 490nm.

### Protein assay

Total protein concentration was estimated with bicinchoninic acid following the procedure described by Smith et al. 1985 [34]. Bovine serum albumin fraction V was used as the standard protein.

### Western blot analysis

Cell lysates from human airways epithelial cells were separated on 4%–12% sodium dodecyl sulfate (SDS) polyacrylamide gels and transferred to polyvinylidene difluoride membranes (PVDF). Immunoblotting was performed using the appropriate antibodies in Tris-based buffered saline with 0.1% Tween 20 and 5% bovine serum albumin. After washing, the membranes were probed with horseradish peroxidase-conjugated goat antiserum to rabbit or mouse. Reactive bands were visualized using chemiluminescence (SuperSignal West Femto; Pierce) on a Kodak 440CF image station. Bands were quantified using Kodak image station software (Kodak 1D 3.6) and ChemiDoc XRS+ System (BIO-RAD). Loading was normalized by probing the membranes with GAPDH antibody.

### Statistical analyses

Continuous data were summarized using means and standard deviations. If data were normal, group differences were tested using t-tests and one-way ANOVAs. The Wilcoxon or Kruskall Wallis tests were used to compare non-parametric distributions between two or more groups to compare group differences. The chi-squared test was used to compare categorical data. Statistical significance was considered at p < 0.05. Unless otherwise specified, statistical analyses were done with Stata 13 (College, TX).

## Results

### Demographics

Human airway epithelial cells were obtained from baseline bronchoscopies from 36 participants of ongoing clinical studies at the University of Colorado. Patients were divided into four groups according to the weight and disease category: a) lean controls (n = 10), b) lean asthmatics (n = 8), c) obese controls (n = 11), and d) obese asthmatics (n = 8). The majority of study participants were female and obese, with moderately controlled asthma (see Table 1).

**Table 1:** Characteristics of the study population. Group differences were tested using t-tests and one-way ANOVAs. Wilcoxon or Kruskall Wallis tests were used to compare non-parametric distributions between two or more groups to compare group differences.

	Lean control (n = 10)	Asthma Lean (n = 8)	Obese control (n = 10)	Asthma Obese (n = 8)	P value
Age years, mean (range)	28 (18–52)	34 (19–58)	30 (19–53)	33 (21–53)	0.6
Sex% (Female)	50%	50%	75%	50%	0.6
BMI (mean, 95% CI)	24 (23–26)	23 (20–26)	35 (31–39)	36 (33–40)	
FEV1% (Mean, 95%CI)	95 (85–104)	91 (83–99)	100 (94–107)	82 (70–94)	0.5
FVC% (Mean, 95%CI)	98 (90–105)	95 (82–108)	102 (95–109)	90 (78–103)	0.4
FEV1/FVC (Mean, 95%CI)	81 (77–85)	80 (71–88)	82 (77–86)	75 (71–78)	.2
eNO ppb (Median Q1–Q3)	15 (10–28)	14 (9–42)	14 10–22)	15.5 (11–23)	0.9
ICS%	NA	50%	NA	78%	NA

BMI: Body Mass Index, FEV1%: Forced exhalation volume in 1 second, % predicted.

FVC: Forced vital capacity % predicted; eNO: exhaled nitric oxide part per billion; ICS: % using inhaled steroids.

P = Oneway ANOVA or Kwallis.

### Primary human airway epithelial cells from obese asthmatics have lower PON2 levels

**Fig 1:**
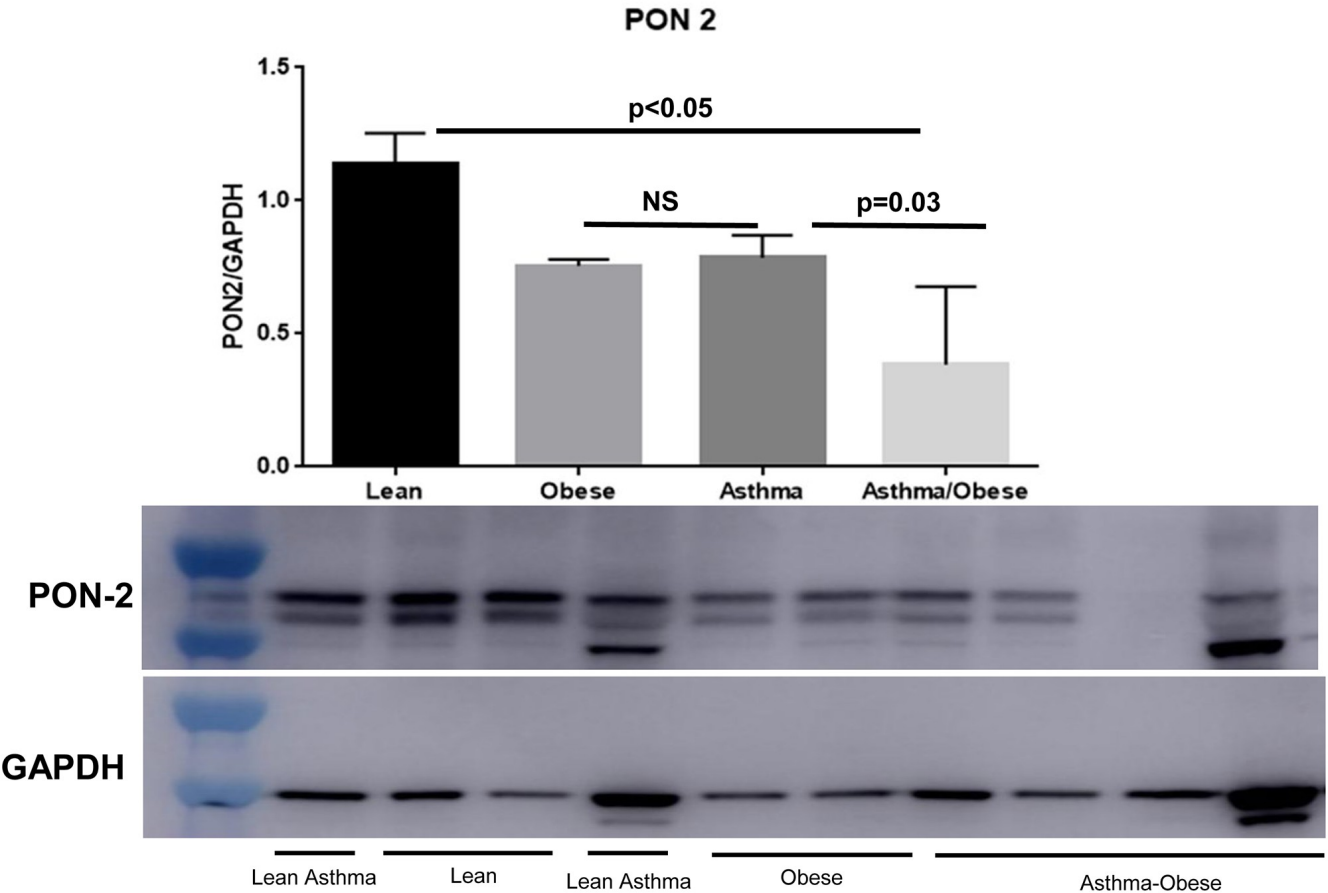
PON2 protein levels in human bronchial epithelial cells from obese and non-obese asthmatics and control subjects. Human bronchial epithelial cells from obese-asthmatics subjects had the lowest PON2 levels when compared with healthy controls. Densitometry of PON2 *p<0.05, N = 4/group one-way ANOVA.

We measured the expression of PON2 using Western Blot in human bronchial epithelial cells obtained from bronchoscopies from obese asthmatics and controls (Table 1). The anti-PON2 antibody detected 2 bands with small differences in molecular weight, showing the presence of two isoforms of PON2. These data are in accordance with previous studies of PON2 [16–18] and the two PON2 mRNA splice variants found in public bioinformatics databases [19]. Compared to lean controls, airway epithelial cells from obese asthmatics had significantly lower PON2 protein levels. Moreover, across weight categories, obese asthmatics had the lowest levels (Fig 1).

### PON2 gene expression exhibits similar trends to the PON2 protein levels

**Fig 2:**
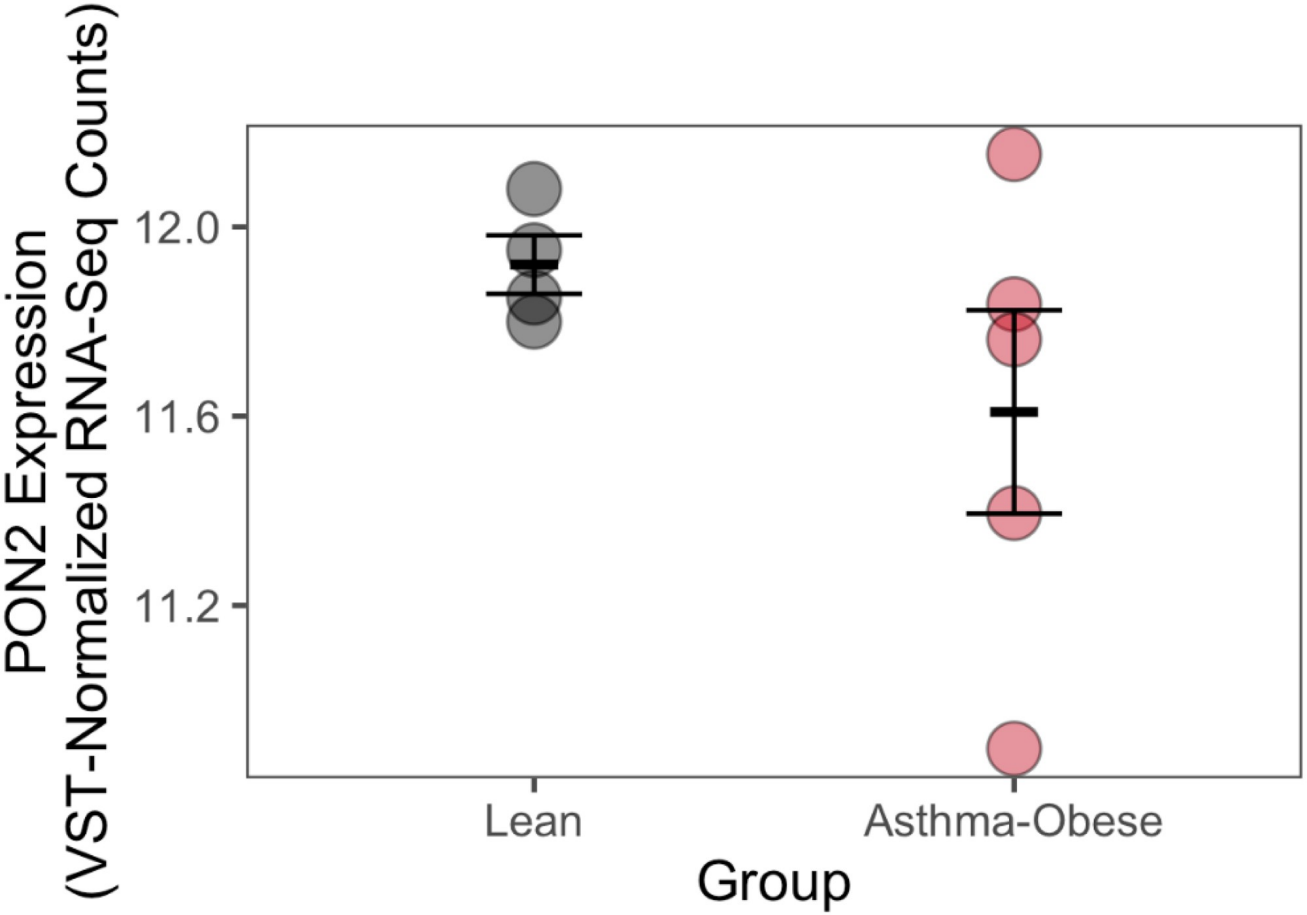
PON2 gene expression exhibits similar trends to the PON2 protein concentration levels measured using Western blot, with a trend towards decreased PON expression in asthma-obese (n = 5, 11.6 ± 0.22; mean ± standard error in VST-normalized messenger RNA levels) compared to lean participants (n = 4, 11.9 ± 0.062). The error bars depicit mean ± standard error.

We measured mRNA levels of PON2 on similarly recruited samples using untargeted RNA-seq [33]. Using these independent measurement method and independent samples, we found that PON2 normalized expression exhibits trends similar to the PON2 protein concentration levels measured using Western blot (Fig 2), although the effect was not statistically significant (DESeq2, P = 0.26).

### PON2 localizes to mitochondria in primary human bronchial epithelial cells

**Fig 3:**
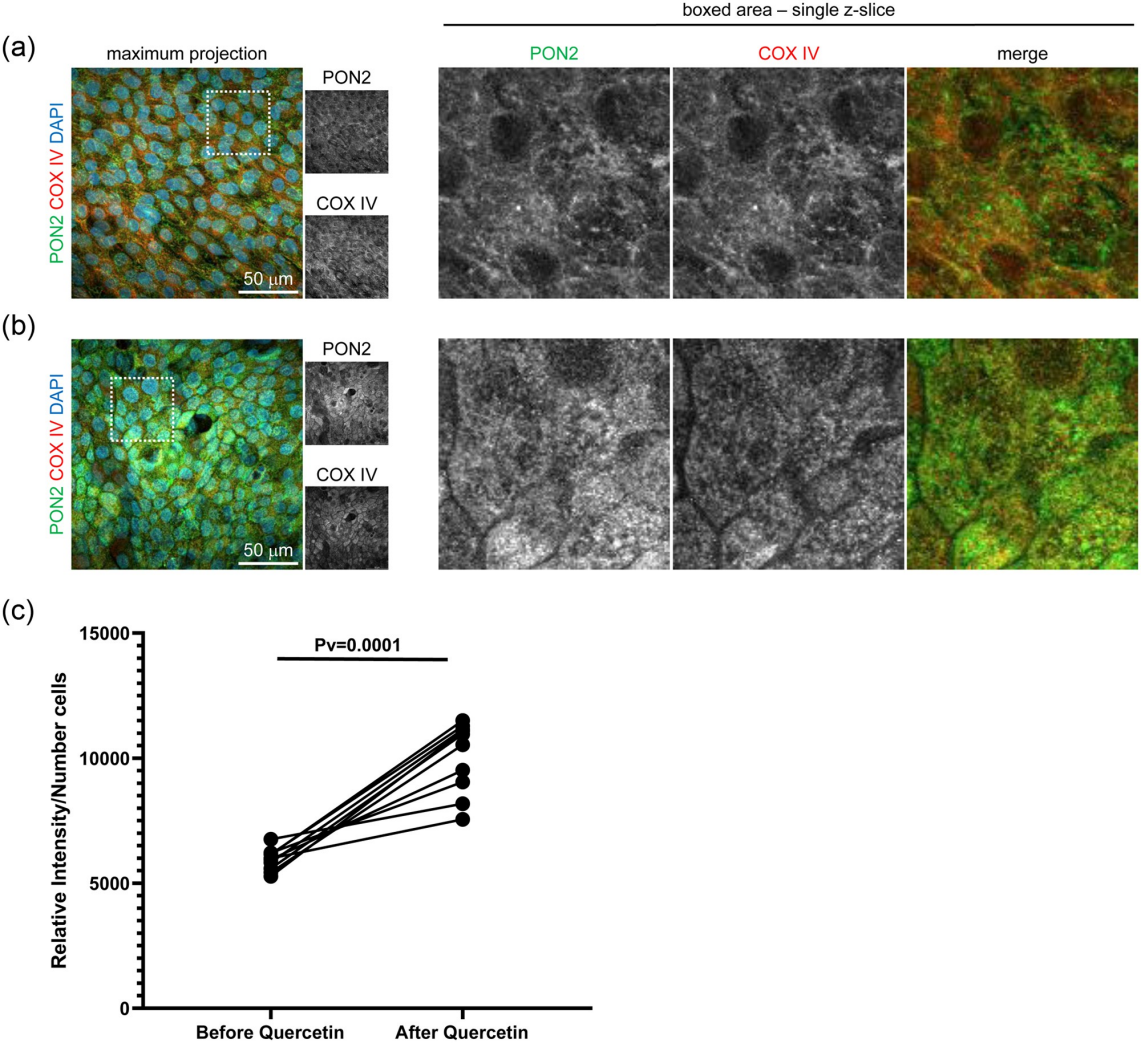
PON2 localization in HBECs. Maximum projection of confocal images stacks (left) of primary HBECs labeled with PON2 (green) and COX IV (red) antibodies and stained with DAPI to mark nuclei. PON2 localizes to mitochondria in primary human bronchial epithelial cells (a), Quercetin treatment does not shift PON2 mitochondria localization and increases its expression in human bronchial epithelial cells (b). Relative intensity of immunolabeling images showed that quercetin treatment on HBEC cultures increases approximately 50% PON2 protein levels (c).

**Fig 4:**
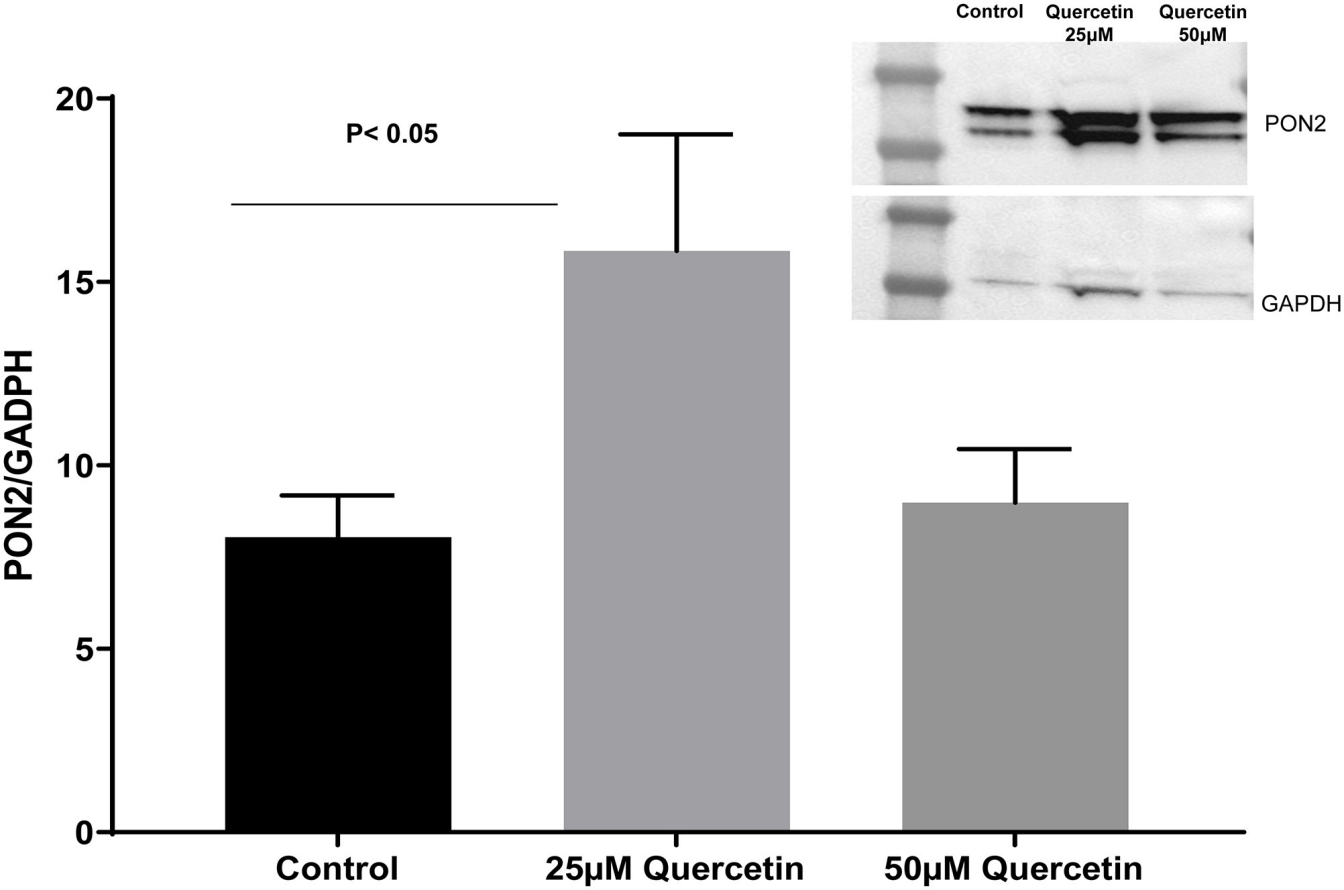
Ciliated, mucus-producing primary airway epithelial cells, maintained for 21 days in air-liquid interface (ALI) were treated with 25 μM or 50μM of quercetin. 25 μM quercetin significantly increased PON2 protein levels compared to control. Densitometry of PON2 *p<0.005, N = 3/group one-way ANOVA.

Consistent with known patterns of localization, immunolabeling with PON2 antibody showed partially overlapping localization to mitochondria in fully differentiated Human Bronchial Epithelial Cells (HBEC) cultures (Fig 3a). While it was detected in all cells, some cells had markedly higher levels of PON2, which suggests a previously unknown airway epithelial cell type specific PON2 expression. In addition, quercetin treatment did not alter the ability PON2 to localize to mitochondria (Fig 3b). Consistent with our finding with protein levels (Fig 4) treatment with 25 μM quercetin on HBEC cultures showed increases of PON2 protein levels (Fig 3c).

### Quercetin significantly augments PON2 levels

Whereas 25 μM quercetin significantly increased PON2 protein levels in comparison to controls, the 50μM did not (Fig 4). This is a well characteristic of biphasic dose-response known as “hormesis,” where an agent produces beneficial or stimulatory effect at low doses but is toxic at higher doses [35, 36]. Quercetin has been reported to have deleterious effects under certain conditions [37, 38]. Rat primary cerebellar granule neurons exposed to increased concentration of quercetin in vitro showed a maximal beneficial effect at 25μM and toxic effect at 50μM and higher doses [35]. Similar beneficial effect of quercetin concentrations have been reported in other studies [23, 39]; therefore, we conducted all subsequent in vitro experiments using 25μM.

### Quercetin treatment reduces cellular and mitochondrial oxidative stress in primary human bronchial epithelial cells

**Fig 5:**
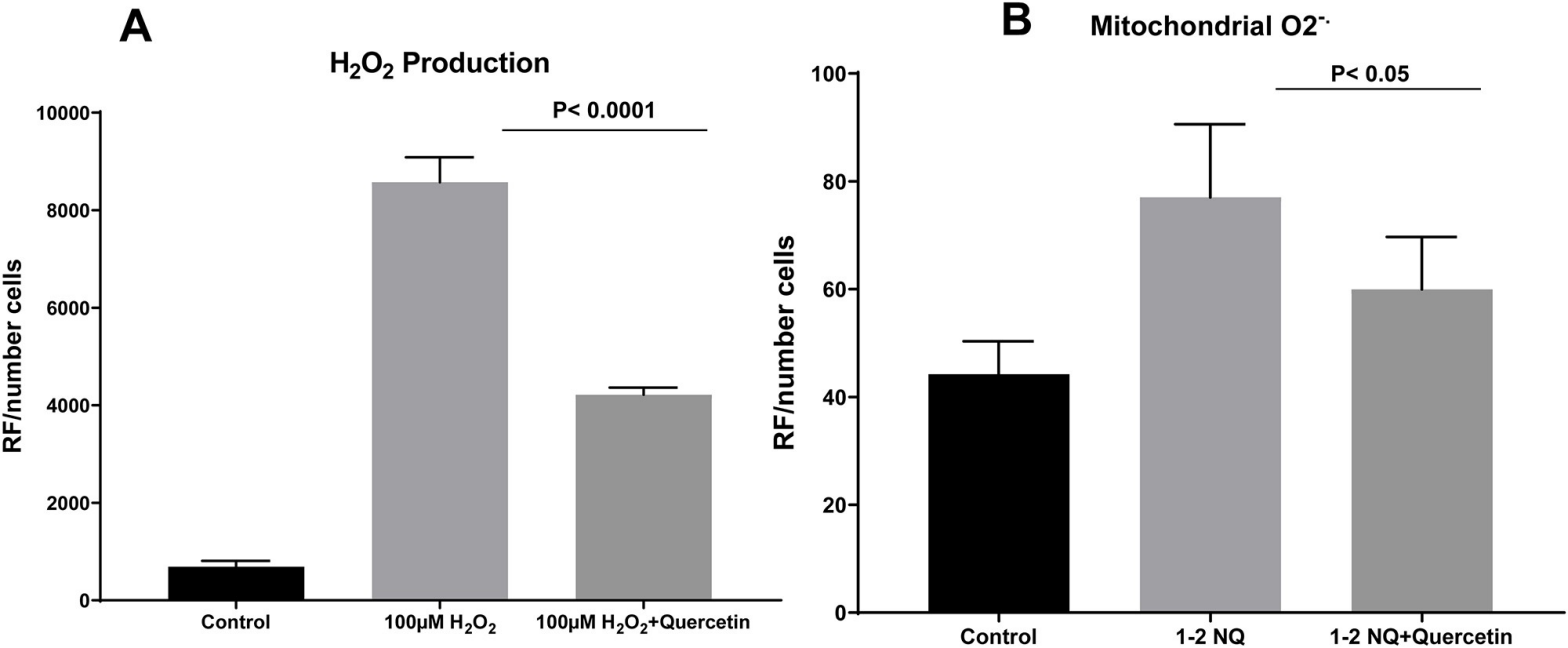
Quercetin reduces cellular and mitochondrial oxidative stress in human airway epithelial cells. Human bronchial epithelial cells in ALI state, control, and treated with 25μM quercetin. A) H_2_O_2_ production p<0.0001, N = 4. B) Mitochondrial ROS production p<0.05, N = 5 one-way ANOVA.

To determine whether quercetin protects from oxidative stress, we exposed human bronchial epithelial cells to two well-known oxidants: hydrogen peroxide (H_2_O_2_) and 1–2 naphthoquinone, which induce mitochondrial ROS production [40]. We found that compared to untreated cells, human bronchial epithelial cells previously treated with 25μM quercetin for 24hrs produced significantly lower levels of hydrogen peroxide and mitochondrial ROS after the oxidant challenge (Fig 5).

### Effect of quercetin in cytotoxicity

**Fig 6:**
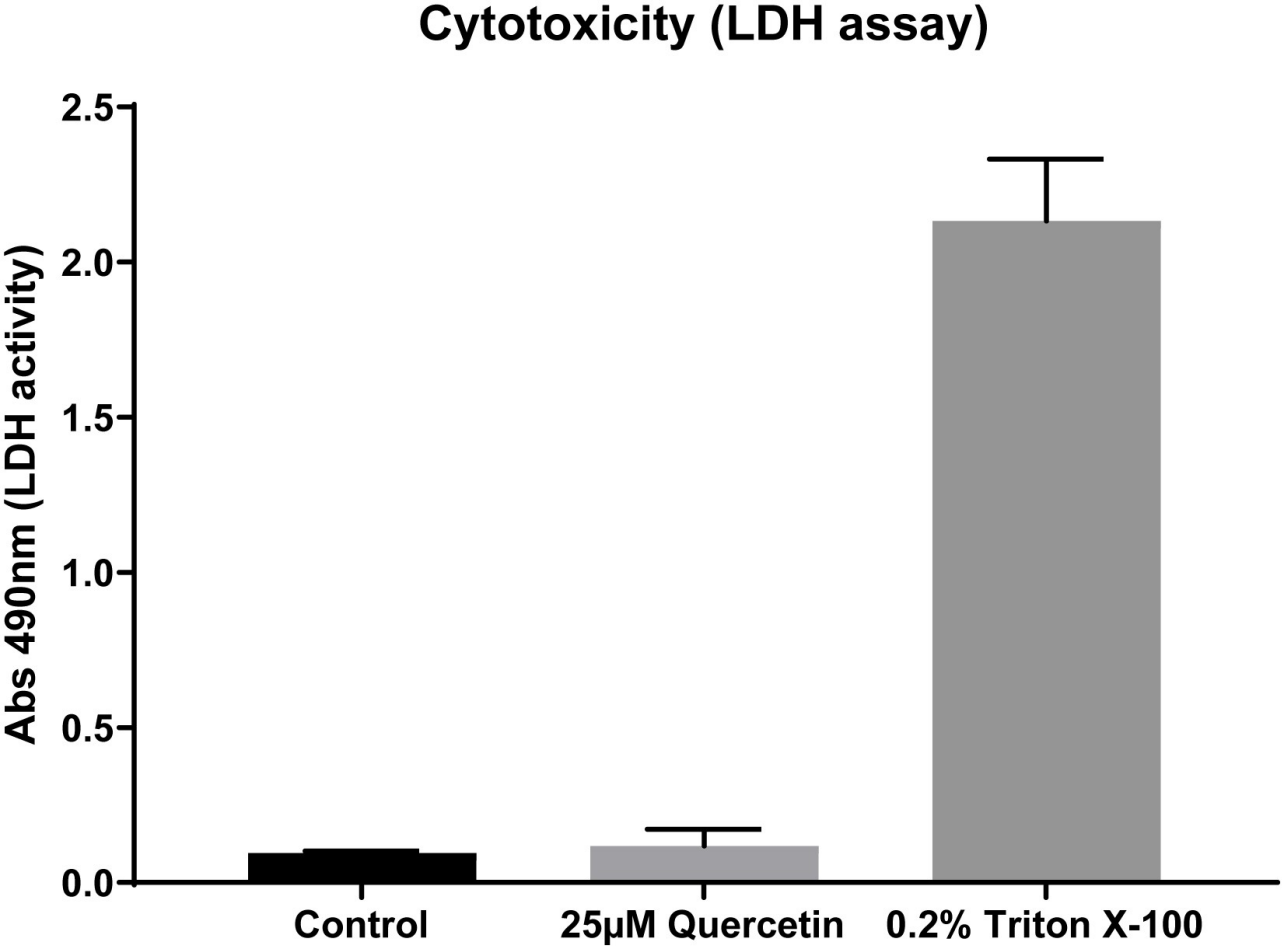
Quercetin cytotoxicity: Human bronchial epithelial cells in ALI state untreated (Control), treated with 25μM quercetin and positive control (Triton X-100, 0.2%), N = 9/group one-way ANOVA.

In concordance with other reported studies [23, 35, 39], we found that 25 μM quercetin supplementation on human bronchial epithelial cells cultures did not induce cell injury as measured by changes in lactic dehydrogenase levels (Fig 6).

### The knockdown of PON2 rendered the cells more susceptible to oxidative stress

**Fig 7:**
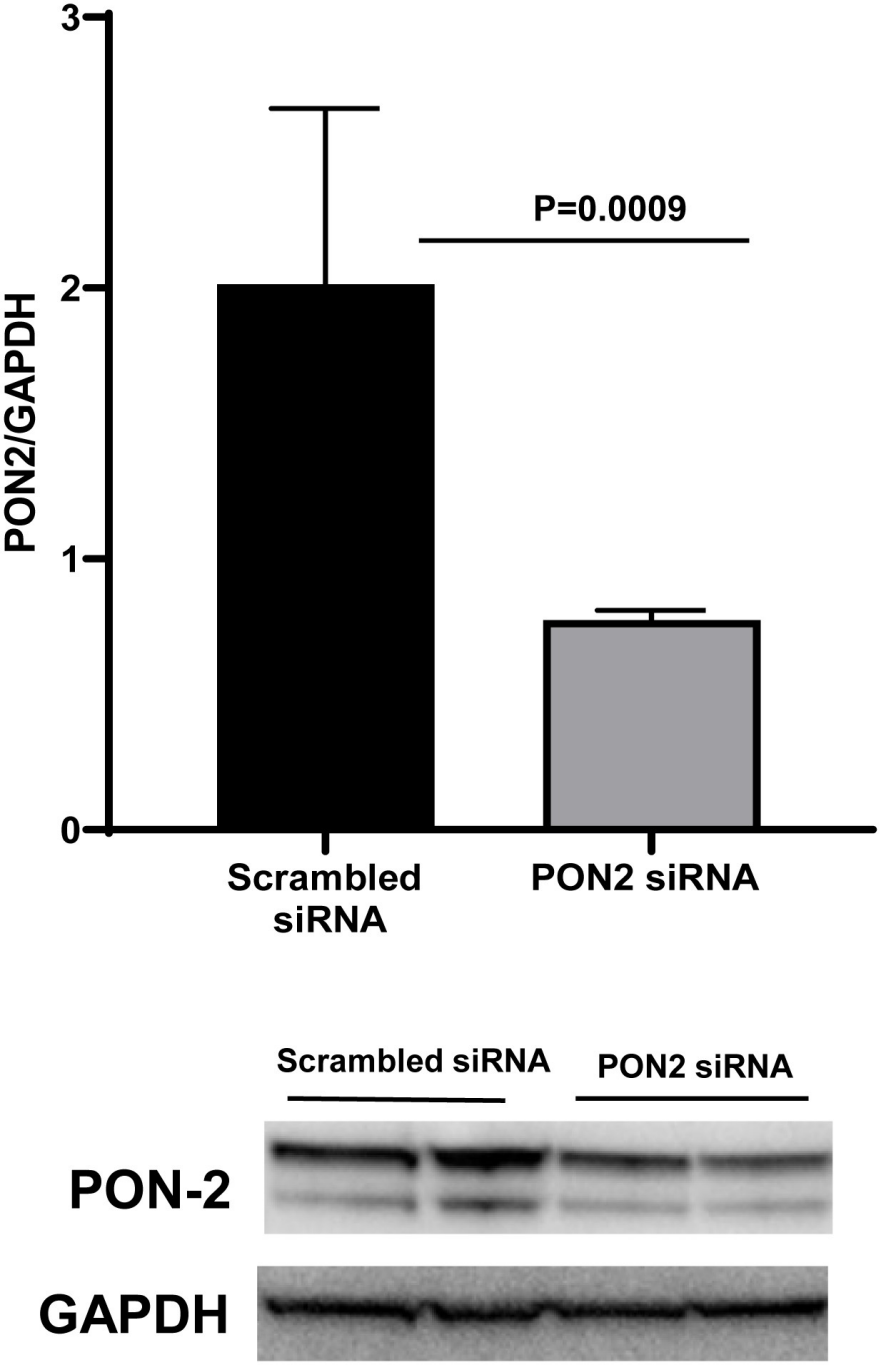
Paraoxonase 2(PON2) knockdown Human Bronchial Epithelial cells (BEAS-2B). At 48h post-transfection, the expression of PON2 were significantly decreased in PON2 knockdown cells. Relative quantification values were determined relative to scrambled siRNA-treated controls cells and ratio of PON2: GAPDH bands was calculated (N = 6) p<0.0009 Unpaired t test.

**Fig 8:**
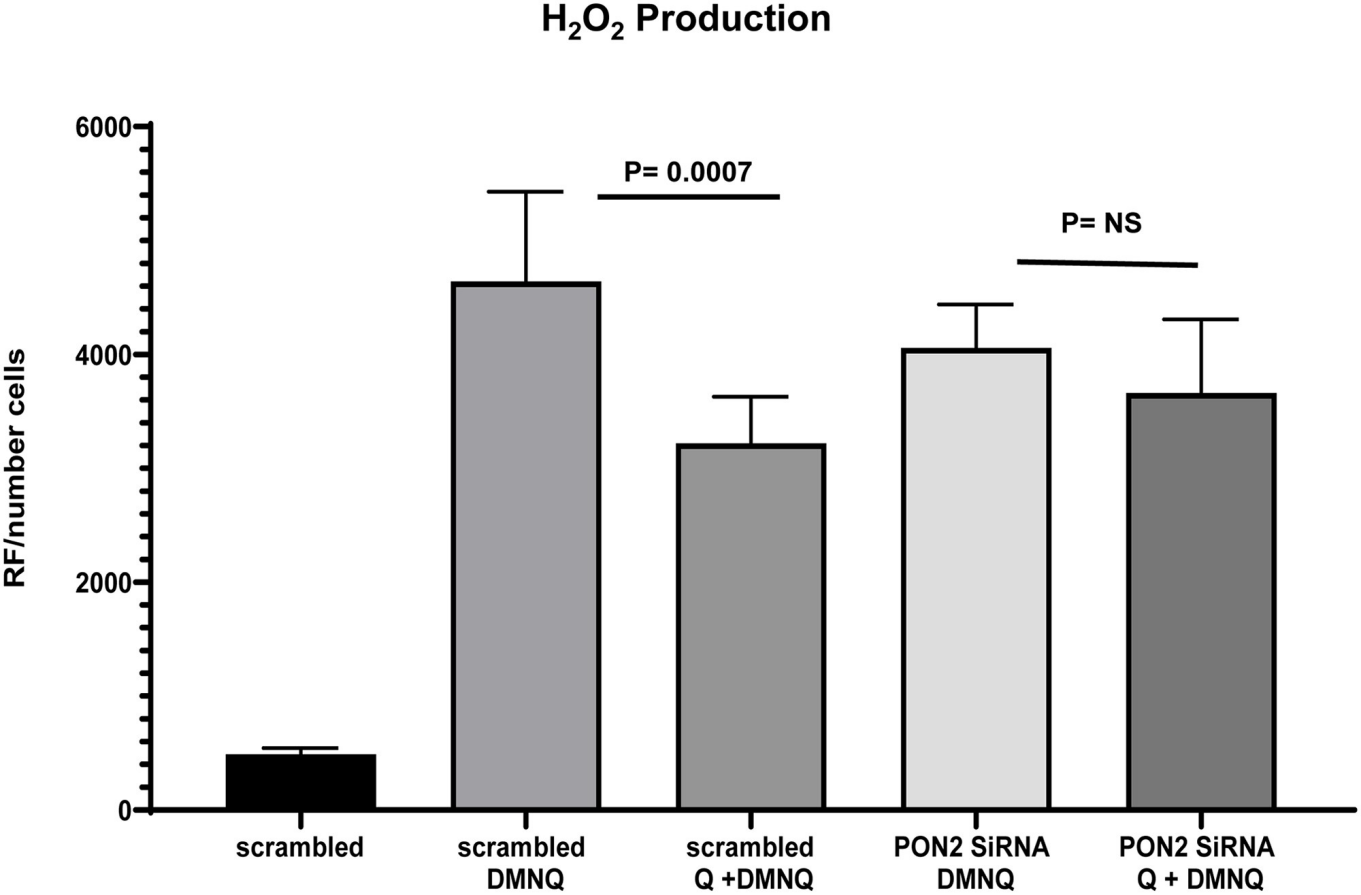
PON2 knockdown rendered cells more susceptible to oxidative stress. The oxidative stress response in siRNA transfected BEAS2B cells treated with quercetin. BEAS2B transfected cells were pretreated for 24 hs with 25μM quercetin. After washout, cells were exposed for 20 minutes to 25μM DMNQ. H_2_O_2_ production was measured by Amplex Red p<0.0007, N = 5/group.one-way ANOVA.

We performed a siRNA transfection study to examine the role of PON2 in modulating BEAS2B’s response to oxidative stress (Fig 7). We exposed siRNA transfected BEAS2B cells to 2,3-Dimethoxy-1,4-naphthoquinone (DMNQ), which induce ROS production. We found that scrambled siRNA cells pretreated with 25μM quercetin for 24hrs showed significantly lower levels of hydrogen peroxide after the oxidant exposure. In contrast, no significant protection was observed in the PON2 siRNA cells pretreated with quercetin (Fig 8).

## Discussion

To our knowledge, the current study is the first one to report the presence of PON2 in mitochondrial of human bronchial airways epithelial cells and its relationship between asthma and obesity. Our results suggest that decreased PON2 protein levels in primary human epithelial cells could potentially be a mechanism contributing to the increased airway oxidative stress in obese asthmatics. Moreover, the findings from this study show that quercetin increases PON2 protein levels reducing oxidative injury in primary asthmatic airway epithelial cells. Interestingly, no significant protection with supplementation of quercetin was observed in PON2 siRNA cells, suggesting that PON2 induction by quercetin represents a critical component of its protective effect, possibly shared by other polyphenols.

Paraoxonase 2 (PON2) is a member of the paraoxonase family of genes, which also includes Paraoxonase 1 (PON1) and Paraoxonase 3 (PON3) [37]. PON2 is an intracellular enzyme primarily restrained in the inner mitochondrial membrane, connected with respiratory Complex III [15]; thus, it has a potential role in protecting cells from excessively produced mitochondrial reactive oxygen species. PON2 has been shown to have potent antioxidant and anti-inflammatory properties in the murine central nervous system, macrophages, and human vascular cells [15–18]. Its central localization in the inner mitochondrial membrane—a significant source of free radical production—suggests an essential role in preventing cells from mitochondrial-derived oxidative damage [15]. This concept is supported by multiple recent papers establishing that PON2 protects against oxidative stress, improves mitochondrial function and diminishes ROS production [41–43]. Here we show for the first time that PON2 localizes to mitochondria in in fully differentiated human epithelial cells cultures and contributes to the cells less susceptible to oxidative stress. Consequently, increasing PON2 expression could have therapeutic potential for obese asthmatics, in whom mitochondrial dysfunction is an essential driver of oxidative stress and increased airway inflammation.

Quercetin is a naturally occurring flavonoid polyphenol present in several plants and fruits. A wide range of biological activities of quercetin has been mentioned in different studies, and most of these activities are attributed to antioxidant properties. Studies have shown that dietary polyphenols can regulate mitochondrial redox status and improve mitochondrial function in both in vitro and in vivo experiments [22]. In addition, quercetin increases PON2 levels in murine macrophages and astrocytes in vitro and restores corticosteroid sensitivity in peripheral blood mononuclear cells (PBMCs) from COPD patients [25]. In murine neuronal cells, the protective effect of quercetin is highly reduced, though not completely abolished by the absence of PON2 [38], this finding provides strong support for a crucial role of PON2 induction in the protection afforded by quercetin.

Quercetin’s mechanism of action is pleitropic. It involves the C-Jun-N-terminal Kinases pathway (JNK), which is activated by a variety of stimuli, such as oxidative stress, and phosphorylates various transcription factors leading to changes in gene expression (3844). Recently it has been reported that quercetin induces a very low-level of oxidative stress, which in turn activates the JNK/AP-1 pathway [44], which increases PON2 expression. On the other hand, it has been shown that estradiol supplementation in vitro on astrocytes and neurons from male mice increased expression of PON2 [45]; therefore, quercetin may induce PON2 expression by virtue its estrogenic activity [46, 47]. In vitro studies with U251 cells shown that quercetin has the potential to cross the blood-brain barrier restrain proliferation and trigger apoptosis [48]. Within in vivo models, upon administration of quercetin at nanomolar levels, quercetin was found in brain tissue, though bioavailability of quercetin can be increased [49].

Interestingly, the formulation of quercetin in nanoparticles significantly increases its penetration in the brain [50, 51]. Nevertheless, oral quercetin in vivo has been shown to protect rodents from oxidative stress and neurotoxicity induced by a variety of agents [52–54]. The ability of quercetin to affect intracellular levels of PON2 is an important future avenue of investigation.

A significant limitation that must be considered when interpreting the results of this study is that they show an "in vitro" effect of quercetin, which should be confirmed in vivo. However, our data suggested that increasing airway PON2 could be a novel therapeutic approach to improve the health of obese asthmatics, who in general, have greater airway oxidative stress, respond less to steroids, and more difficult to control. These results support quercetin as a novel therapeutic agent that could safely improve the health of obese subjects with asthma.

## Conclusion

This is the first study to highlight the importance of PON2 as an antioxidant mechanism in primary human airway epithelial cells and its potential pathophysiologic role in mitochondrial dysfunction. Our findings suggest that decreased PON2 protein levels in human airways epithelial cells contribute to increased oxidative stress in obese asthmatics. Further work is needed to translate these findings into clinical relevance by testing whether quercetin supplementation can lessen obesity-mediated airway oxidative stress in asthmatics. If successful, these data will lead to the implementation of more considerable phenotype-driven and precision-based phase II proof of concept studies. Currently, there are no pharmacologic strategies to reduce airway oxidative stress in obese asthmatics.

## Supporting information

S1 Fig PON2 protein levels in human bronchial epithelial cells. Data relevant to densitometry of PON2 *p<0.05, N = 4/group one-way ANOVA.
(XLSX)

S2 Fig PON2 normalized gene expression. Normalized data from RNAseq (Lean N = 4; Asthma-obese N = 5).
(XLSX)

S3 Fig PON2 localization in HBECs. Data relevant to Relative Intensity of PON2 expression.
(XLSX)

S4 Fig Human bronchial epithelial cells in ALI state untreated and treated with 25μM and 50μM quercetin. Densitometry data.
(XLSX)

S5 Fig Oxidative stress reduced by 25μM quercetin in human bronchial epithelial cells. Data of relative fluorescence normalized by number cells, N = 5 one-way ANOVA.
(XLSX)

S6 Fig Quercetin cytotoxicity: Human bronchial epithelial cells in ALI state untreated, treated with 25μM quercetin and positive control. LDH activity data (Abs 490nm), N = 9/group one-way ANOVA.
(XLSX)

S7 Fig Paraoxonase 2(PON2) knockdown Human Bronchial Epithelial cells (BEAS-2B). Densitometry data, (N = 6) p<0.0009 Unpaired t test.
(XLSX)

S8 Fig Oxidative stress response in PON2 knockdown BEAS2B cells. Data of relative fluorescence normalized by number cells, N = 5/group.one-way ANOVA.
(XLSX)

S1 Data (XLSX)

S1 Raw images (PDF)
