# Supplementary material for: Airway epithelial Paraoxonase-2 in obese asthma
Source: PLoS One. 2022 Mar 14;17(3):e0261504. doi: 10.1371/journal.pone.0261504 (PMC8920196; doi:10.1371/journal.pone.0261504)
Supplement: S1 Raw images — (PDF) [file pone.0261504.s010.pdf]

Figure 1: PON2

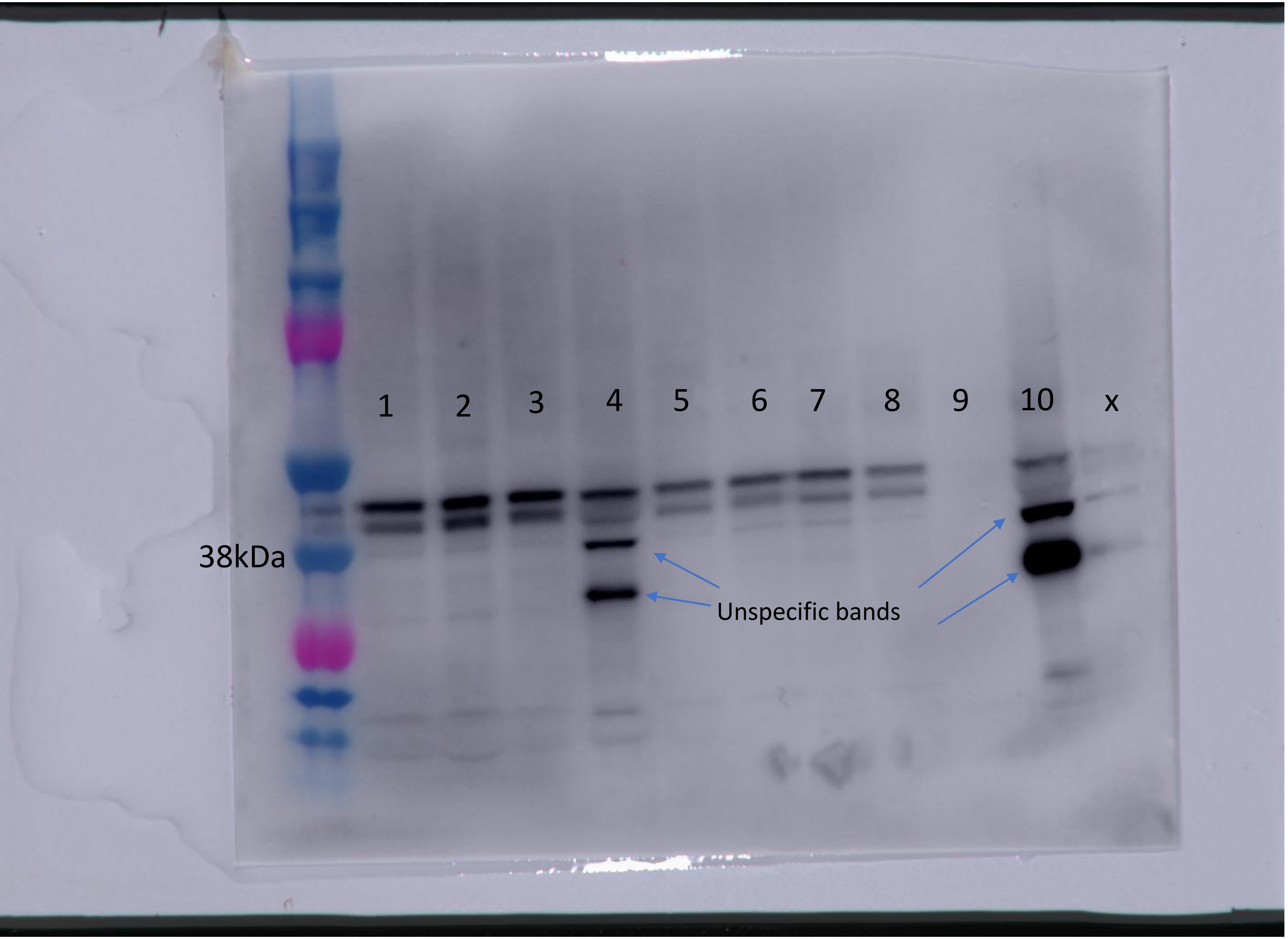

Reactive bands were visualized using chemiluminescence (SuperSignal West Femto; Pierce) on a Kodak 440CF image station. Bands were quantified using Kodak image station software (Kodak 1D 3.6).

Lean: 2,3

Lean Asthma: 1,4

Obese: 5,6

Asthma Obese: 7,8,9,10

Figure 1: GAPDH

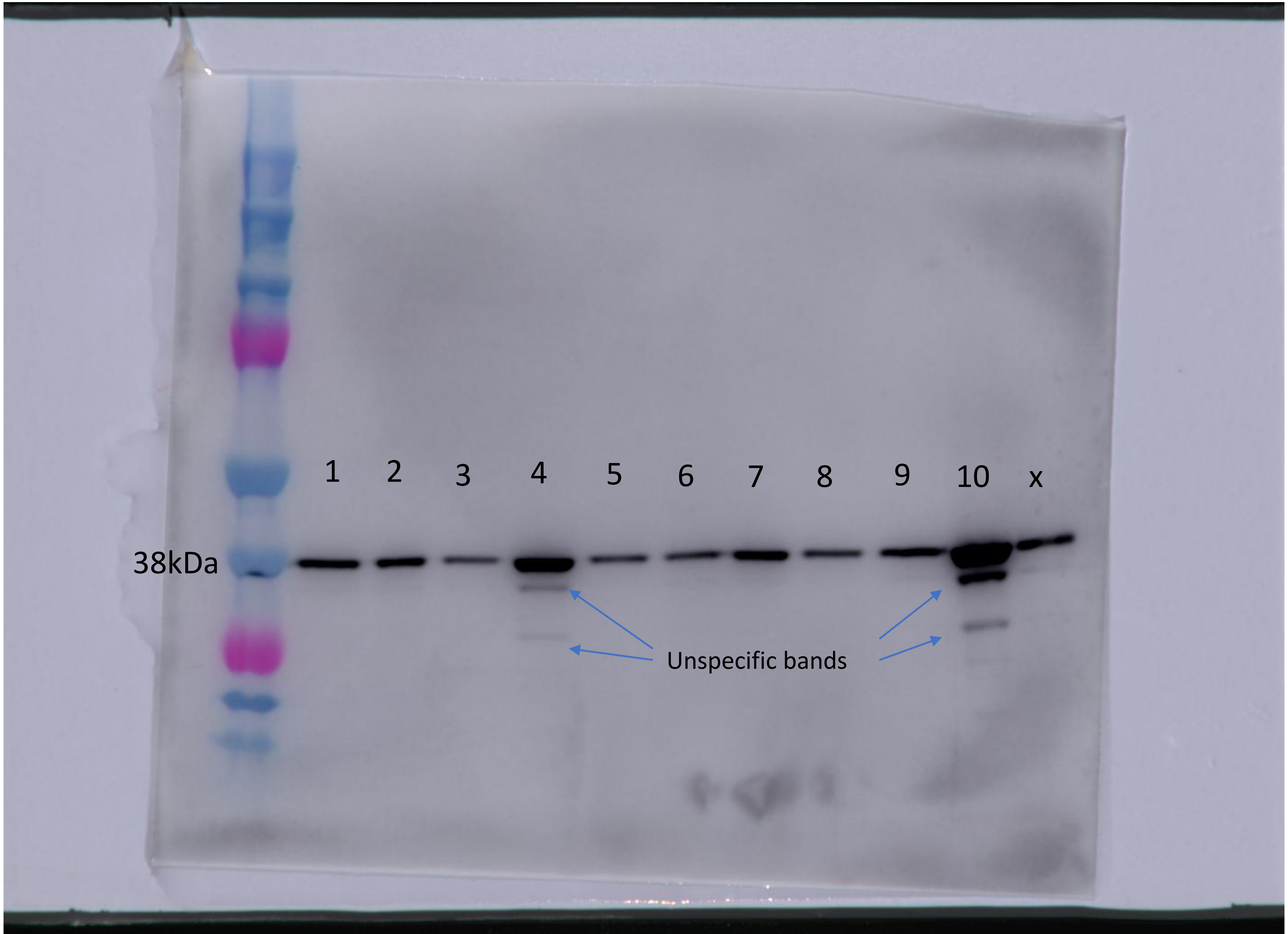

Reactive bands were visualized using chemiluminescence (SuperSignal West Femto; Pierce) on a Kodak 440CF image station. Bands were quantified using Kodak image station software (Kodak 1D 3.6).

Lean: 2,3

Lean Asthma: 1,4

Obese: 5,6

Asthma Obese: 7,8,9,10

Figure 4: PON2

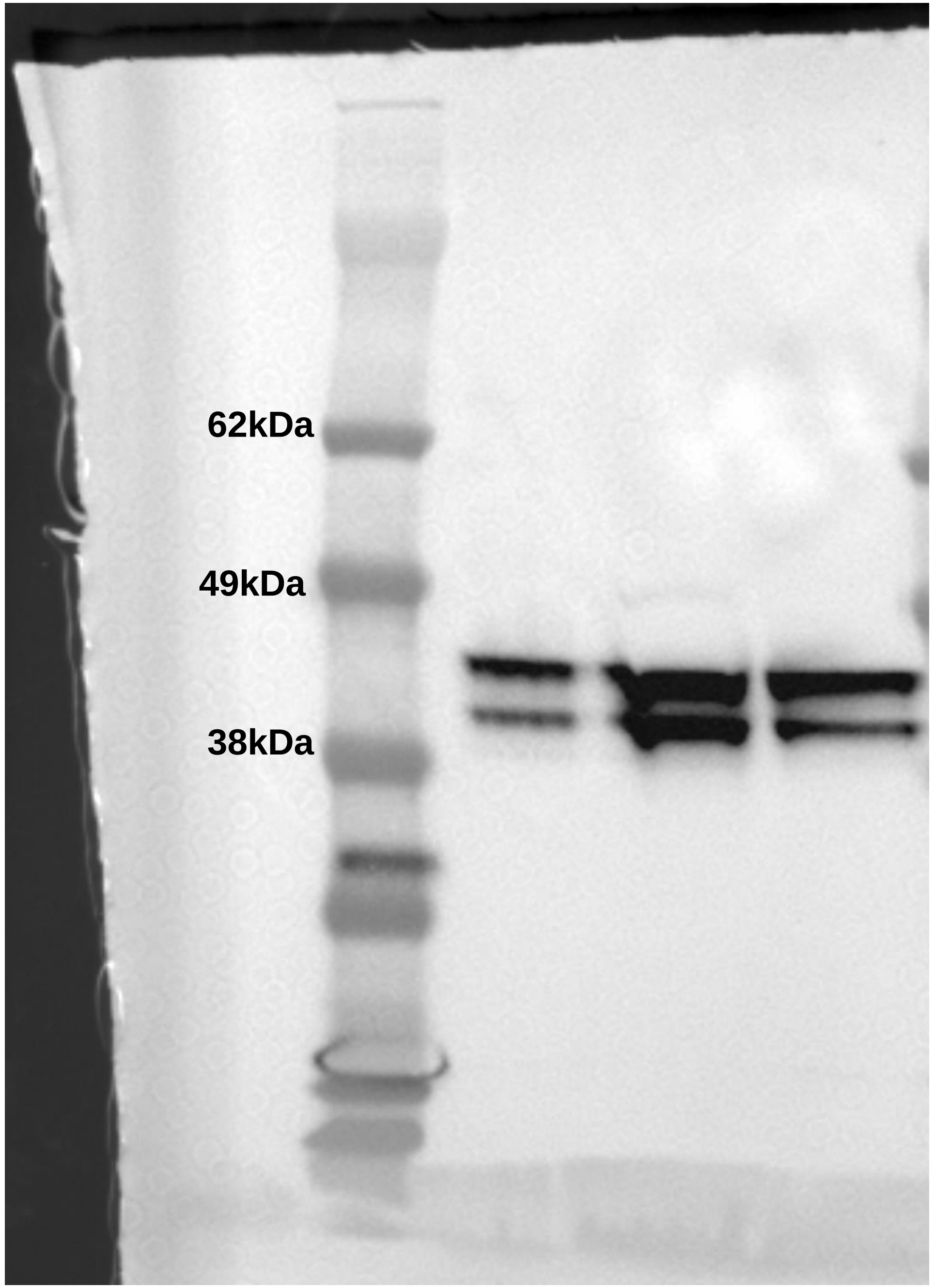

Reactive bands were visualized using chemiluminescence (SuperSignal West Femto; Pierce) on ChemiDoc XRS+ System.

Figure 4 : GAPDH

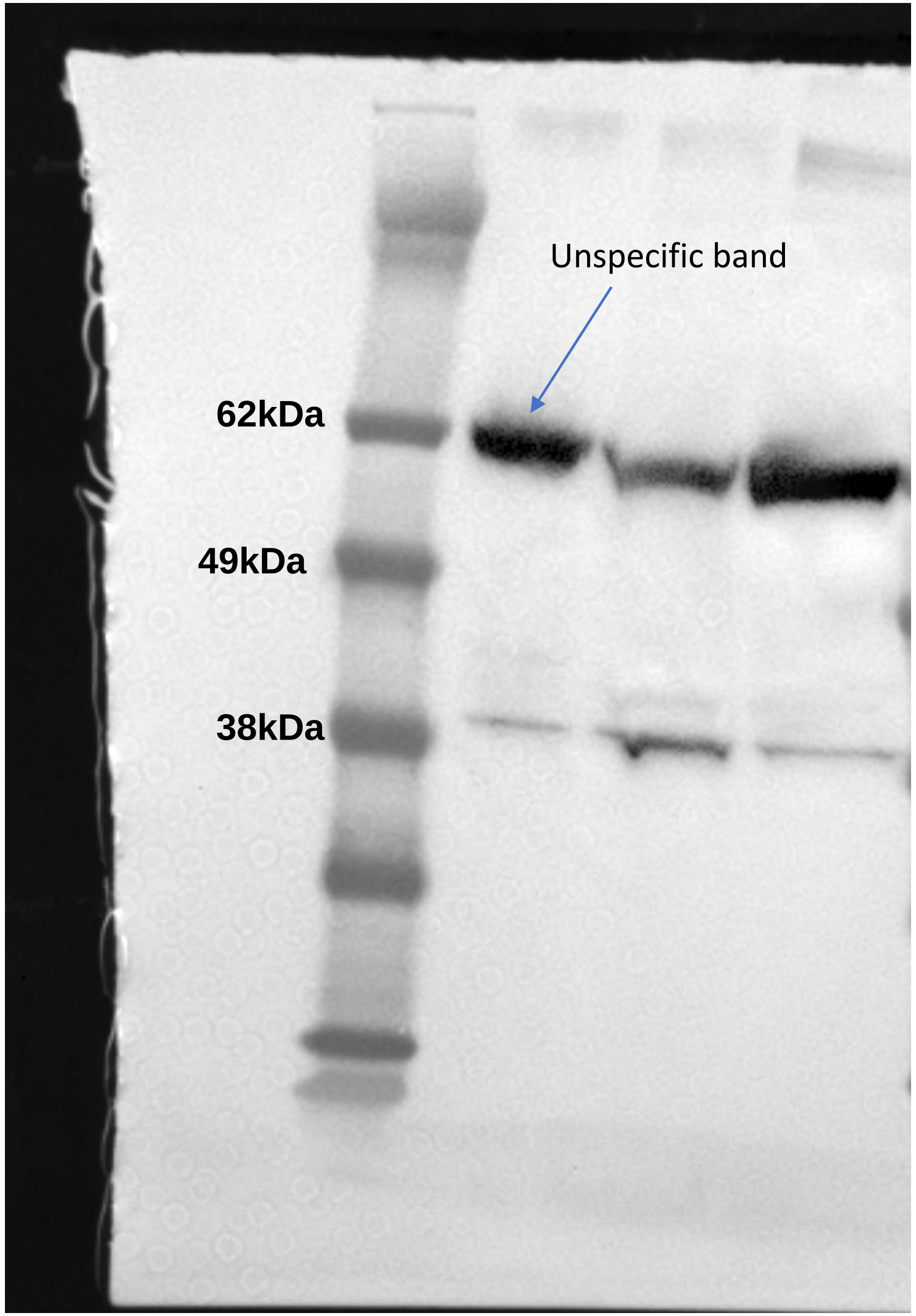

Reactive bands were visualized using chemiluminescence (SuperSignal West Femto; Pierce) on ChemiDoc XRS+ System.

Figure 7: PON2

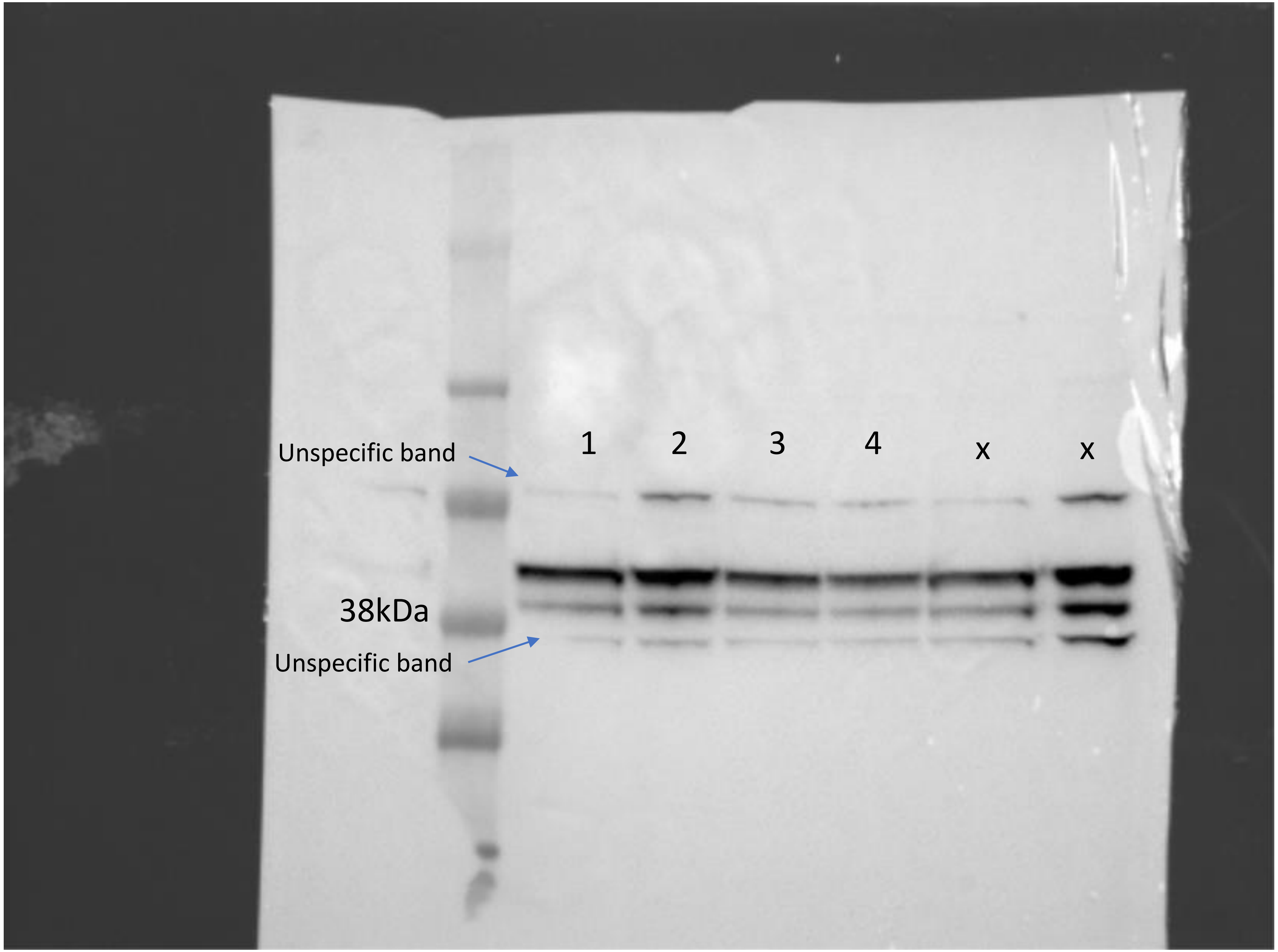

Reactive bands were visualized using chemiluminescence (SuperSignal West Femto; Pierce) on ChemiDoc XRS+ System

1: scrambled siRNA

2: scrambled siRNA

3: PON2 siRNA

4: PON2 siRNA

Figure 7:GAPDH

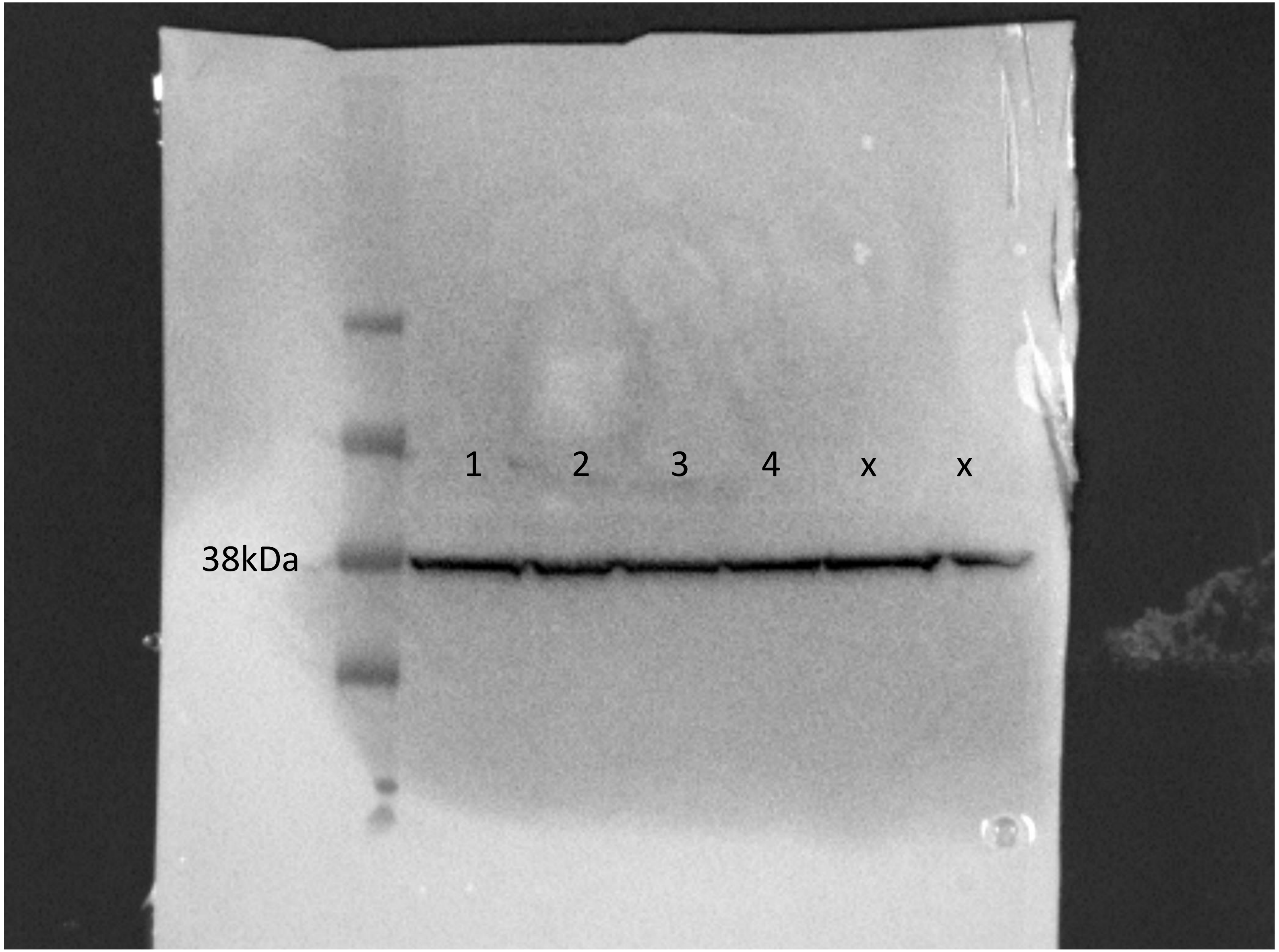

Reactive bands were visualized using chemiluminescence (SuperSignal West Femto; Pierce) on ChemiDoc XRS+ System.

1: scrambled siRNA

2: scrambled siRNA

3: PON2 siRNA

4: PON2 siRNA
